# Epidemiological and spatial characteristics of interpersonal physical violence in a Brazilian city: A comparative study of violent injury hotspots in familial versus non-familial settings, 2012-2014

**DOI:** 10.1371/journal.pone.0208304

**Published:** 2019-01-07

**Authors:** Kevan Guilherme Nóbrega Barbosa, Blake Byron Walker, Nadine Schuurman, Sérgio D’avila Lins Bezerra Cavalcanti, Efigênia Ferreira e Ferreira, Raquel Conceição Ferreira

**Affiliations:** 1 Universidade Federal de Minas Gerais, Department of Community and Preventive Dentistry, Belo Horizonte-, Brazil; 2 Geographisches Institut, Humboldt-Universität zu Berlin, Berlin, Germany; 3 Department of Geography, Simon Fraser University, Burnaby BC, Canada; 4 Universidade Estadual da Paraíba, Department of Dentistry, Campina Grande, Brazil; Syracuse University, UNITED STATES

## Abstract

This study explores both epidemiological and spatial characteristics of domestic and community interpersonal violence. We evaluated three years of violent trauma data in the medium-sized city of Campina Grande in North-Eastern Brazil. 3559 medical and police records were analysed and 2563 cases were included to identify socioeconomic and geographic patterns. The associations between sociodemographic, temporal, and incident characteristics and domestic violence were evaluated using logistic regression. Using Geographical Information Systems (GIS), we mapped victims’ household addresses to identify spatial patterns. We observed a higher incidence of domestic violence among female, divorced, or co-habitant persons when the violent event was perpetrated by males. There was only a minor chance of occurrence of domestic violence involving firearms. 8 out of 10 victims of domestic violence were women and the female/male ratio was 3.3 times greater than that of community violence (violence not occurring in the home). Unmarried couples were twice as likely to have a victim in the family unit (OR = 2.03), compared to married couples. Seven geographical hotspots were identified. The greatest density of hotspots was found in the East side of the study area and was spatially coincident with the lowest average family income. Aggressor sex, marital status, and mechanism of injury were most associated with domestic violence, and low-income neighbourhoods were coincident with both domestic and non-domestic violence hotspots. These results provide further evidence that economic poverty may play a significant role in interpersonal, and particularly domestic violence.

## Introduction

Violence is estimated to have caused 475 000 deaths in 2012, with the highest rates concentrated in low- and middle-income countries of the Americas, with an average of 28.5 homicides per 100 000 population [1]. However, mortality represents only a small number of the total population burdened by violence, including family members, friends of victims, and the community [2,3]. According to the WHO, tens of millions of people are victimized by injuries, and violence plays a significant role in this context, resulting in significant impacts on medical programmes, economic costs, mental health, and links to substance abuse [4].

The burden of domestic violence is historically greatest among children, women, and the elderly. The Global Status Report of the World Health Organization (WHO) revealed that a quarter of adults had been victims of physical abuse as children, one-in-three women had been victimized by an intimate partner, and six per cent of elderly persons reported some abuse in the month preceding the WHO’s investigation [1].

Interpersonal violence encapsulates both domestic and community violence; the former includes cases involving family members and people in romantic or otherwise intimate relationships, while the latter occurs between non-related people, who may or may not be mutually anonymous [5]. In many countries, the term *domestic violence* refers to intimate partner violence; this study uses domestic violence to include a broader range of victim-aggressor relationships encompassing child, elder, and partner abuse, and more broadly familial incidents [6].

A large body of literature explores risk factors for violence, broadly categorising drivers into behavioural and environmental. Recent studies have sought to empirically drive a theoretical basis for a broader, more ecological approach, in which behaviours are produced and reproduced through individual experiences of violence (e.g., childhood abuse), socioeconomic and cultural phenomena (e.g., economic marginalisation), and the built environment (e.g., areas of high building and population density in which a large number of human-to-human encounters take place, increasing opportunities for violence).

There is general agreement within the literature that alcohol consumption and the nature of familial or partner relationships are the most significant predictors of familial or intimate-partner violence, compared to which the environment plays no significant direct role [7]. However, an ecological approach, in which human behaviours are influenced by a person’s socioeconomic, cultural, and built environments, facilitates a broader understanding of factors implicit in the production of violent behaviours [8] and societal and individual reactions to the presence of violence [9]. For example, the presence and density of alcohol serving establishments predicts interpersonal violence, particularly in spaces where high-volume alcohol consumption are culturally reinforced (e.g., nightclubs and sports stadia) [10] or reinforced through socioeconomic deprivation [11]. It thereby is theorised that the confluence of multiple built, sociocultural, and economic environmental factors increase the probability of alcohol-related behaviours that may lead to domestic violence.

In Brazil, domestic violence, particularly against women, has been steadily increasing over the last three decades, the rate of female-victim homicide has more than doubled since 1980 and this country presented among the highest recorded rates of violence against women globally, surpassed only by El Salvador, Colombia, Guatemala, and Russia [12]. The Brazilian homicide rate for women is 48 times more than in the United Kingdom, 24 times more than Ireland and Denmark, and 16 times more than Japan and Scotland [12]. Some legislative efforts have been made, such as Brazilian Law nr. 10 778/2003 [13], which requires that all cases of domestic violence against women are reported by medical staff. Law n. 11 340/2006 [14] increased the mandatory judicial punishment for aggressors against women, and Law n. 13 104/2015 [15] that reclassified murder against a female family victim as a heinous crime.

Studies of violence and health in Brazil have, however, mostly focussed on the medical characteristics of injuries. Hospital reports typically include minimal or no information relating to the event. The recent mandatory registration of all familial, sexual, and otherwise violent incidents has resulted in a large governmental database called SINAN (*Sistema de Informação de Agravos de Notificação*), which records all recorded cases of interpersonal violence within Brazil’s Health Services [16].

In response to the pressing need for targeted violence prevention and response, this study aims to map and describe the cases of physical violence in the urban area of Campina Grande, located in Northeastern Brazil, and to evaluate the association between sociodemographic, temporal and incident characteristics of violence. We also include selected socio-epidemiological characteristics of victims and their neighbourhoods to inform violence prevention and response strategies in low- and middle-income countries.

## Data and methods

### Research ethics

This study conforms to Resolution 466/12 of the Brazilian Ministry of Health and the Declaration of Helsinki, and was approved by two independent Human Research Ethics Committees: Universidade Estadual da Paraíba (cert.: 0652.0.133.000–11); and Universidade Federal de Minas Gerais (cert.: 47207815.5.0000.5149).

### Study setting

We examined three years of violent incidents from 2012 to 2014 in Campina Grande, a Brazilian city located in the Northeastern region. The urban area has two public universities, many private colleges, and a large commercial and industrial presence. The study area was home to 402 912 residents in 2014 [17] and is one of the largest cities in Northeastern Brazil, excluding the state capitals. The urban area is divided into north, south, east, and west zones. There are many informal settlements that are unrecognised by the local government. Officially, the city comprised 49 neighbourhoods at the time of this study.

### Incident data

All data were obtained in 2015 from the Forensic Institute of Campina Grande, Brazil. This Forensic Center is a Scientific Civil Police Service wherein the victims of violence are examined by a trauma physician. The reports are then used to assist with criminal proceedings. Before being admitted to the Forensic Center for examination, a victim must file a police report. Victims who do not file a report are therefore not included in this study, as is unfortunately common in studies of interpersonal violence.

### Data extraction and validation

Data extraction was conducted by manually reading trauma reports and subsequently obtaining data regarding variables of interest. Two researchers conducted these procedures following a training session, through which we sought to standardize the search phase. Thereafter, we completed a pilot study to validate the data extraction and digitization protocols. Based on 30 reports from the year 2011 (which were not included in this study), we identified some between-examiner error in event description, and therefore updated and delivered a new training program. The second-phase intra-examiner difference (50 reports from 2012, which were included in this study) resulted in a Kappa score of 0.85, which was considered to be satisfactory for the purposes of this analysis.

Two members of the research team manually digitised 3559 paper reports, corresponding to the total number of violence-related cases between 2012 and 2014. We retained victims’ sociodemographic (household address; sex; age; marital status; education level; occupation; location of violence), temporal (day of week; time of day), and incident characteristic (medical description of the trauma site; victim’s relationship to the aggressor).

### Epidemiologic and statistical analysis

Statistical analyses were performed using SPSS (v. 20). We elected to use an alpha threshold of 0.05 to assess the significance of all associations. Chi-square, binary- and multiple logistic regression were used to assess the association among sociodemographic, temporal, incident characteristics and domestic violence. The final model was constructed by introducing all variables that showed association with domestic violence in the bivariate analysis with *p*<0.25. The variables that were statistically associated with domestic violence were maintained in the final model. The education and location of occurrence were excluded from the multivariable analysis due to the high percentage of missing data.

### Spatial analysis using location of victim’s household address

Geographical Information Systems (GIS) were used to map each victim’s household address and identify hotspots in the study area. Hotspots are areas with the highest concentration of violence victims, which appear as an intense red color in the maps. Each household was mapped as a point using ArcGIS (v.10.4) and the resulting locations were manually validated against the tabular victim records. We excluded 996 cases due to missing/erroneous address information, homeless victims, and cases that took place in the rural periphery of the study area. A total of 2563 cases were successfully mapped (72.01% of total). Census tract data were freely obtained from the website of the Brazilian Institute of Geography and Statistics (IBGE) [18].

We used Kernel Density Estimation (KDE) to identify hotspots of violent injury. This technique is commonly used to identify clusters of point locations, and has been applied and validated in studies of violent trauma [11,19,20].The purpose of the density analysis was to highlight absolute concentrations of victim households, not rates (*per capita*), because resource allocation (e.g., medical services, community social programmes) should generally target areas with the greatest need (the highest concentration of cases). A kernel bandwidth of 300 metres was selected, as this parameter was found to best represent the apparent spatial pattern of points while also approximating the radius of the average neighbourhood in the study area. An output cell size of 10 metres was selected, as this represented the approximate geographical precision of injury points, as evaluated visually and with local knowledge of the study area. To correlate the number of victims and the local population we calculated violent injury rates per square kilometer using census tract populations. The Brazilian census tract is smaller than a neighborhood, which included many census tracts. We used the rates to standardize the hotspot analysis results and then identified and mapped statistically significant clusters of high rates using the Global and Local Moran’s I spatial statistics. The optimal search bandwidth (300 metres) for this function was selected using Ripley’s K function.

We also conducted a descriptive analysis of the hotspots, focussing on victim age and sex, aggressor sex, type of violence, and average family income (per census tract (2010 Brazilian census, [18]) in which the victims were residing at the time of the incident).

## Results

### Epidemiological characteristics

**Fig 1:**
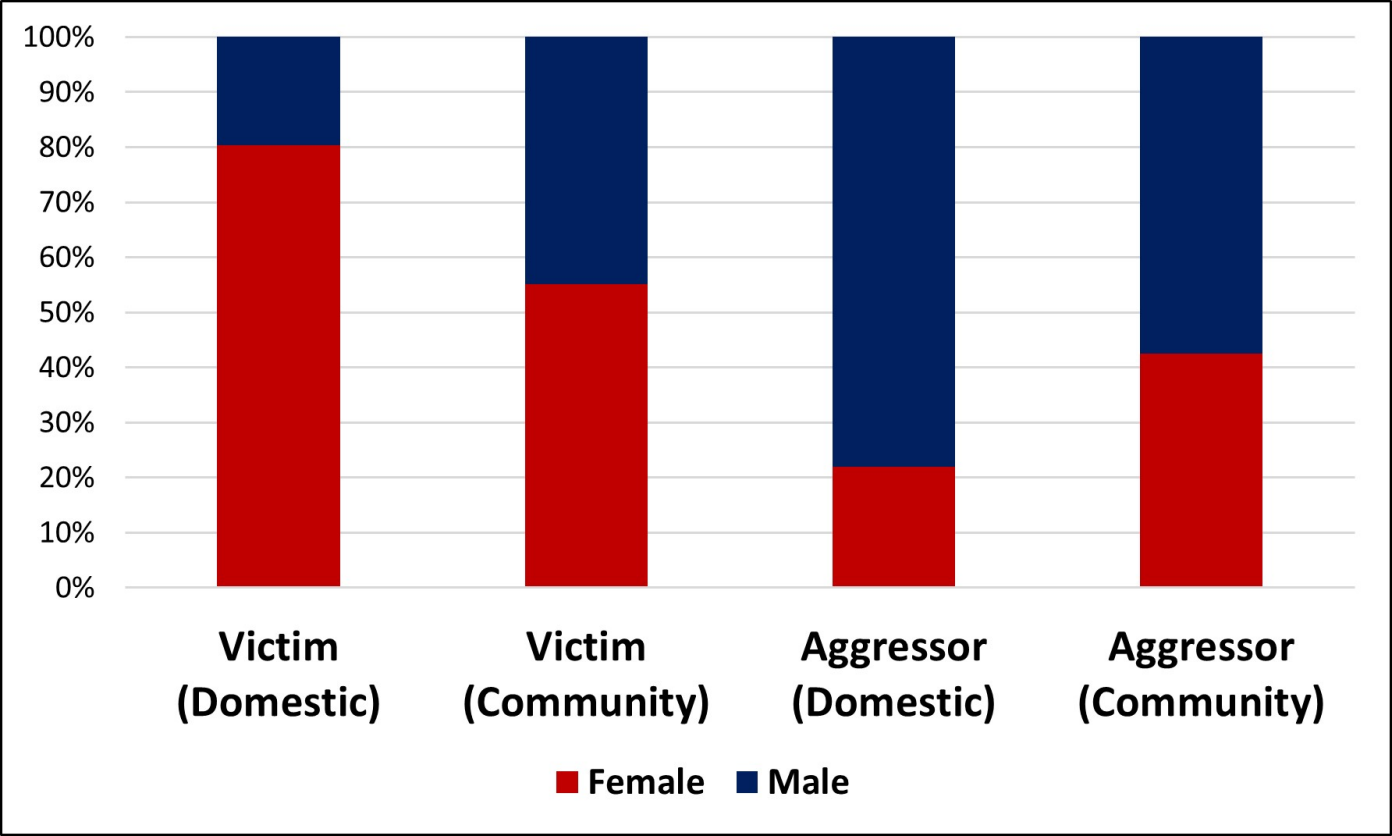
Distribution for sex; victims and aggressors. (χ^2^ *p* < 0.001 for both domestic and community).

**Fig 2:**
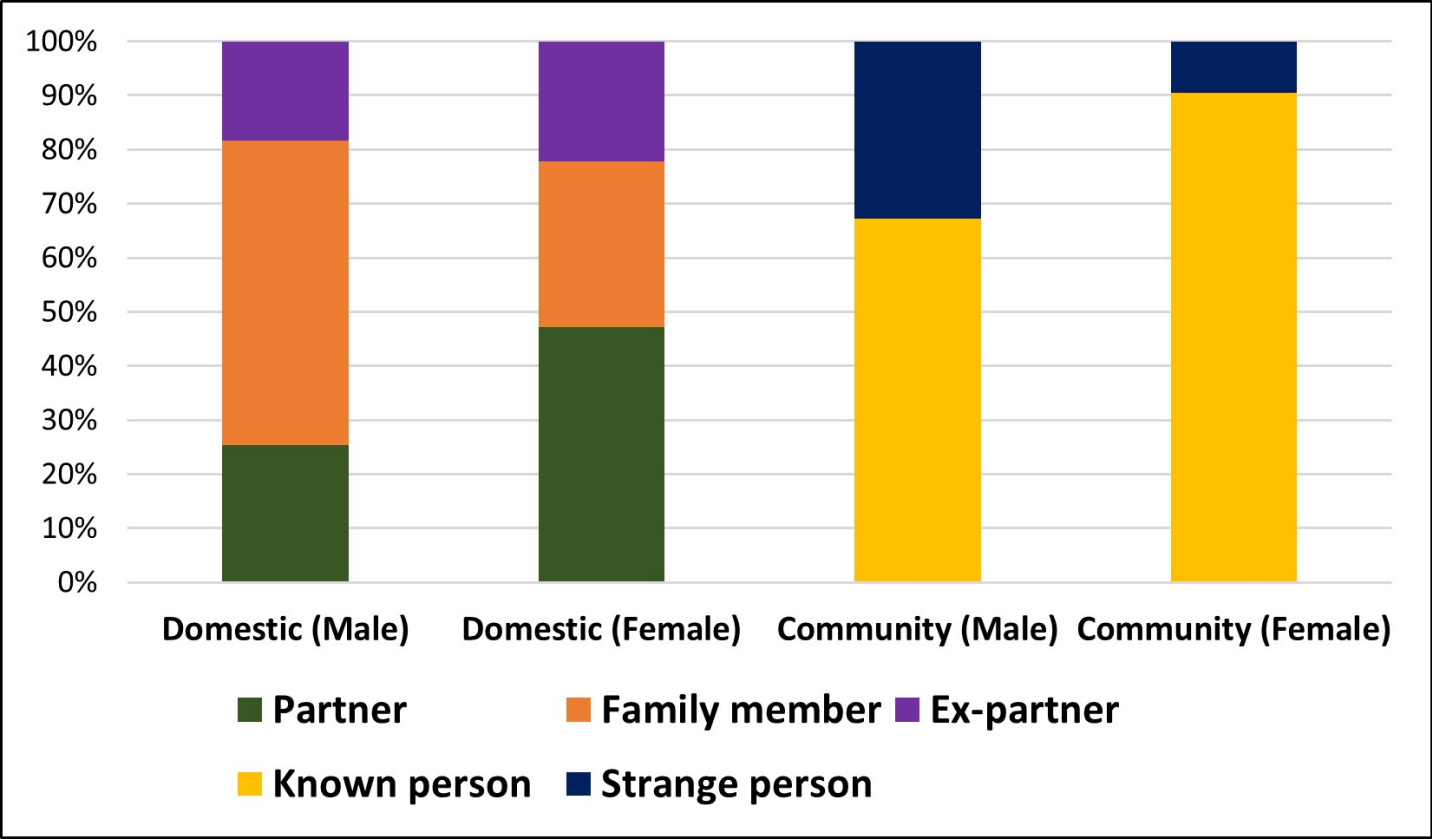
Distribution for aggressor-victim relationship; domestic and community.

Sociodemographic, temporal, and incident characteristics of victims were explored, as reported in Table 1, and Figs 1 and 2. The majority of victims were young working adults, single, with primary school completed. Temporally, we observed a peak of occurrence on Sundays and evenings. Most incident data were related to bodily injury and affected more than one anatomical region.

**Table 1:** Sociodemographic, temporal and incident characteristics among victims of violence. All 2563 cases for Campina Grande urban area, 2012−2014.

	Physical Violence			
	Domestic	Community	Unknown	TOTAL
**TOTAL**	1369 (100%)	1145 (100%)	49 (100%)	2563 (100%)
**SOCIODEMOGRAPHIC VARIABLES**				
**Age−Range**				
young (0−18 years)	220 (16.1%)	241 (21.0%)	9 (18.4%)	470 (18.3%)
young adults (19−39 years)	873 (63.8%)	656 (57.3%)	32 (65.3%)	1561 (60.9%)
middle adults (40−59 years)	231 (16.9%)	205 (17.9%)	7 (14.3%)	443 (17.3%)
older (60 and more)	45 (3.3%)	43 (3.8%%)	1 (2.0%)	89 (3.5%)
**Marital Status**				
single	662 (48.4%)	659 (57.6%)	25 (51.0%)	1346 (52.5%)
married	299 (21.8%)	255 (22.3%)	8 (16.3%)	562 (21.9%)
living together	290 (21.2%)	142 (12.4%)	12 (24.5%)	444 (17.3%)
divorced	84 (6.1%)	52 (4.5%)	1 (2.0%)	137 (5.3%)
widower	26 (1.9%)	17 (1.5%)	1 (2.0%)	44 (1.7%)
unknown	8 (0.6%)	20 (1.7%)	2 (4.1%)	30 (1.2%)
**Education Level**				
unknown information	553 (40.4%)	374 (32.7%)	30 (61.2%)	957 (37.3%)
6−14 years	369 (27.0%)	329 (28.7%)	8 (16.3%)	706 (27.5%)
15−17 years	299 (21.8%)	293(25.6%)	10 (20.4%)	602 (23.5%)
18/more	124 (9.1%)	133 (11.6%)	1 (2.0%)	258 (10.1%)
no literate	24 (1.8%)	16 (1.4%)	-	40 (1.6%)
**Occupation**				
wage earner	452 (33.0%)	394 (34.4%)	14 (28.6%)	860 (33.6%)
autonomous	288 (21.0%)	299 (26.1%)	17 (34.7%)	604 (23.6%)
student	287 (21.0%)	283 (24.7%)	11 (22.4%)	581 (22.7%)
not working*	285 (20.8%)	116 (10.1%)	4 (8.2%)	405 (15.8%)
retired	24 (1.8%)	28 (2.4%)	1 (2.0%)	53 (2.1%)
unknown information	21 (1.5%)	18 (1.6%)	2 (4.1%)	41 (1.6%)
unemployed	12 (0.9%)	7 (0.6%)	-	19 (0.7%)
**Location of Violence**				
inside home	589 (43.0%)	153 (13.4%)	6 (12.2%)	748 (29.2%)
outside home	300 (21.9%)	776 (67.8%)	17 (34.7%)	1093 (42.6%)
unknown information	480 (35.1%)	216 (18.9%)	26 (18.9%)	722 (28.2%)
**TEMPORAL VARIABLES**				
**Day of Week**				
monday	176 (12.9%)	140 (12.2%)	6 (12.2%)	322 (12.6%)
tuesday	159 (11.6%)	138 (12.1%)	5 (10.2%)	302 (11.8%)
wednesday	157 (11.5%)	142 (12.4%)	3 (6.1%)	302 (11.8%)
thursday	150 (11.0%)	112 (9.8%)	4 (8.2%)	266 (10.4%)
friday	156 (11.4%)	149 (13.0%)	11 (22.4%)	316 (12.3%)
saturday	230 (16.8%)	189 (16.5%)	7 (14.3%)	426 (16.6%)
sunday	319 (23.3%)	265 (23.1%)	12 (24.5%)	596 (23.3%)
unknown information	22 (1.6%)	10 (0.9%)	1 (2.0%)	33 (1.3%)
**Time of Day**				
dawn	116 (8.5%)	110 (9.6%)	9 (18.4%)	235 (9.2%)
morning	215 (15.7%)	204 (17.8%)	7 (14.3%)	426 (16.6%)
afternoon	381 (27.8%)	337 (29.4%)	7 (14.3%)	725 (28.3%)
night	534 (39.0%)	415 (36.2%)	13 (26.5%)	962 (37.5%)
unknown information	123 (9.0%)	79 (6.9%)	13 (26.5%)	215 (8.4%)
**INCIDENT CHARACTERISTICS**				
**Mechanism of Injury**				
bodily force	939 (68.6%)	753 (65.8%)	8 (16.3%)	1700 (66.3%)
others	151 (11.0%)	149 (13.0%)	4 (8.2%)	304 (11.9%)
unknown information	141 (10.3%)	95 (8.3%)	8 (16.3%)	244 (9.5%)
mixed	90 (6.6%)	48 (4.2%)	1 (2.0%)	139 (5.4%)
sharp object	41 (3.0%)	55 (4.8%)	8 (16.3%)	104 (4.1%)
firearm	7 (0.5%)	45 (3.9%)	20 (40.8%)	72 (2.8%)
**Body Region Injured**				
more than one	711 (51.9%)	574 (50.1%)	21 (42.9%)	1306 (51.0%)
upper limb	278 (20.3%)	221 (19.3%)	11 (22.4%)	510 (19.9%)
face	208 (15.2%)	199 (17.4%)	4 (8.2%)	411 (16.0%)
lower limb	73 (5.3%)	58 (5.1%)	7 (14.3%)	138 (5.4%)
head	42 (3.1%)	33 (2.9%)	2 (4.1%)	77 (3.0%)
chest	18 (1.3%)	25 (2.2%)	3 (6.1%)	46 (1.8%)
neck	15 (1.1%)	16 (1.4%)	-	31 (1.2%)
back	18 (1.3%)	12 (1.0%)	-	30 (1.2%)
abdomen	6 (0.4%)	6 (0.5%)	1 (2.0%)	13 (0.5%)
unknown information	-	1 (0.1%)	-	1 (0.0%)

*Including female homemaker, preschool kids and pension receivers.

Fig 1 shows a great number of female victims of domestic violence, while male aggressors represent the majority of community violence incidents. In domestic violence, the aggression by an intimate partner was very high. In community violence, the aggression perpetrated by a person unknown to the victim was higher for male victims compared to female (Fig 2).

Odds ratios representing the chance for domestic violence were shown in Table 2. The final logistic model included the following significant variables: victim sex; marital status; mechanism of injury; and aggressor sex. There was a higher chance of occurrence of domestic violence among female victims who were either divorced or living together unmarried, with a male aggressor using multiple weapons/bodily force. A small number of domestic violence cases involved firearms. These associations were independent of the age of the victims.

**Table 2:** Results of the binary and multiple regression with the adjusted model.

	Binary Regression				Multiple Regression (Adjusted Model)			
Domestic Violence	OR	CI-95%		p-value	OR	CI-95%		p-value
		Lower	Upper			Lower	Upper	
**Victim’s Sex**								
male	1	1	1		1	1	1	
female	3.32	2.78	3.96	< 0.001^a^	3.24	2.61	4.05	< 0.001^b^
**Age−Range**								
0−18 y	1	1	1		1	1	1	
19−39 y	1.45	1.18	1.79	< 0.001^a^	1.23	0.89	1.69	0.20
40−59 y	1.23	0.95	1.60	0.11	1.01	0.68	1.51	0.92
60/more	1.14	0.72	1.80	0.55	1.26	0.65	2.46	0.48
**Marital Status**								
single	1	1	1		1	1	1	
widower	1.52	0.81	2.83	0.18	1.73	0.80	3.73	0.15
divorced	1.60	1.11	2.31	0.01^a^	2.02	1.29	3.17	0.002^b^
married	1.16	0.95	1.42	0.12	1.25	0.97	1.61	0.08
living together	2.03	1.61	2.55	< 0.001^a^	1.76	1.34	2.33	< 0.001^b^
**Occupation**								
wage earner	1	1	1		1	1	1	
autonomous	0.84	0.68	1.03	0.10	0.95	0.74	1.21	0.70
retired	0.74	0.42	1.31	0.30	0.68	0.32	1.43	0.31
unemployed	1.49	0.58	3.83	0.40	1.50	0.52	4.32	0.45
not working*	2.14	1.66	2.76	< 0.001^a^	1.35	1.01	1.82	0.04^b^
student	0.88	0.71	1.09	0.25	1.09	0.78	1.52	0.58
**Mechanism of Injury**								
bodily force	1	1	1		1	1	1	
firearm	0.12	0.05	0.27	< 0.001^a^	0.17	0.07	0.40	< 0.001^b^
sharp object	0.59	0.39	0.90	0.01^a^	0.66	0.42	1.04	0.07
others	0.81	0.63	1.03	0.09	0.96	0.73	1.27	0.79
mixed	1.50	1.04	2.16	0.02^a^	2.01	1.33	3.06	0.001^b^
**Aggressor’s Sex**								
female	1	1	1		1	1	1	
male	2.62	2.20	3.12	< 0.001^a^	2.89	2.37	3.53	< 0.001^b^
**Body Region**								
more than one	1	1	1		1	1	1	
head	1.02	0.64	1.64	0.91	-	-	-	
face	0.84	0.67	1.05	0.13	-	-	-	
neck	0.75	0.37	1.54	0.44	-	-	-	
chest	0.58	0.31	1.07	0.08	-	-	-	
abdomen	0.80	0.25	2.51	0.71	-	-	-	
back	1.21	0.57	2.53	0.61	-	-	-	
upper limb	1.01	0.82	1.25	0.88	-	-	-	
lower limb	1.01	0.70	1.45	0.93	-	-	-	
**Day of Week**								
Sunday	1	1	1		1	1	1	
Monday	1.04	0.79	1.37	0.75	-	-	-	
Tuesday	0.95	0.72	1.26	0.75	-	-	-	
Wednesday	0.91	0.69	1.21	0.55	-	-	-	
Thursday	1.11	0.82	1.49	0.47	-	-	-	
Friday	0.87	0.65	1.14	0.32	-	-	-	
Saturday	1.01	0.78	1.30	0.93	-	-	-	
**Time of Day**								
morning	1	1	1		1	1	1	
dawn	1.00	0.72	1.38	0.99	-	-	-	
afternoon	1.07	0.84	1.36	0.56	-	-	-	
night	1.22	0.97	1.53	0.09	-	-	-	

^a^Significant at 5% (binary regression).

^b^Significant at 5% (multiple regression).

*Including female homemaker, preschool kids and pension receivers.

#### Spatial analysis: Hotspots

**Fig 3:**
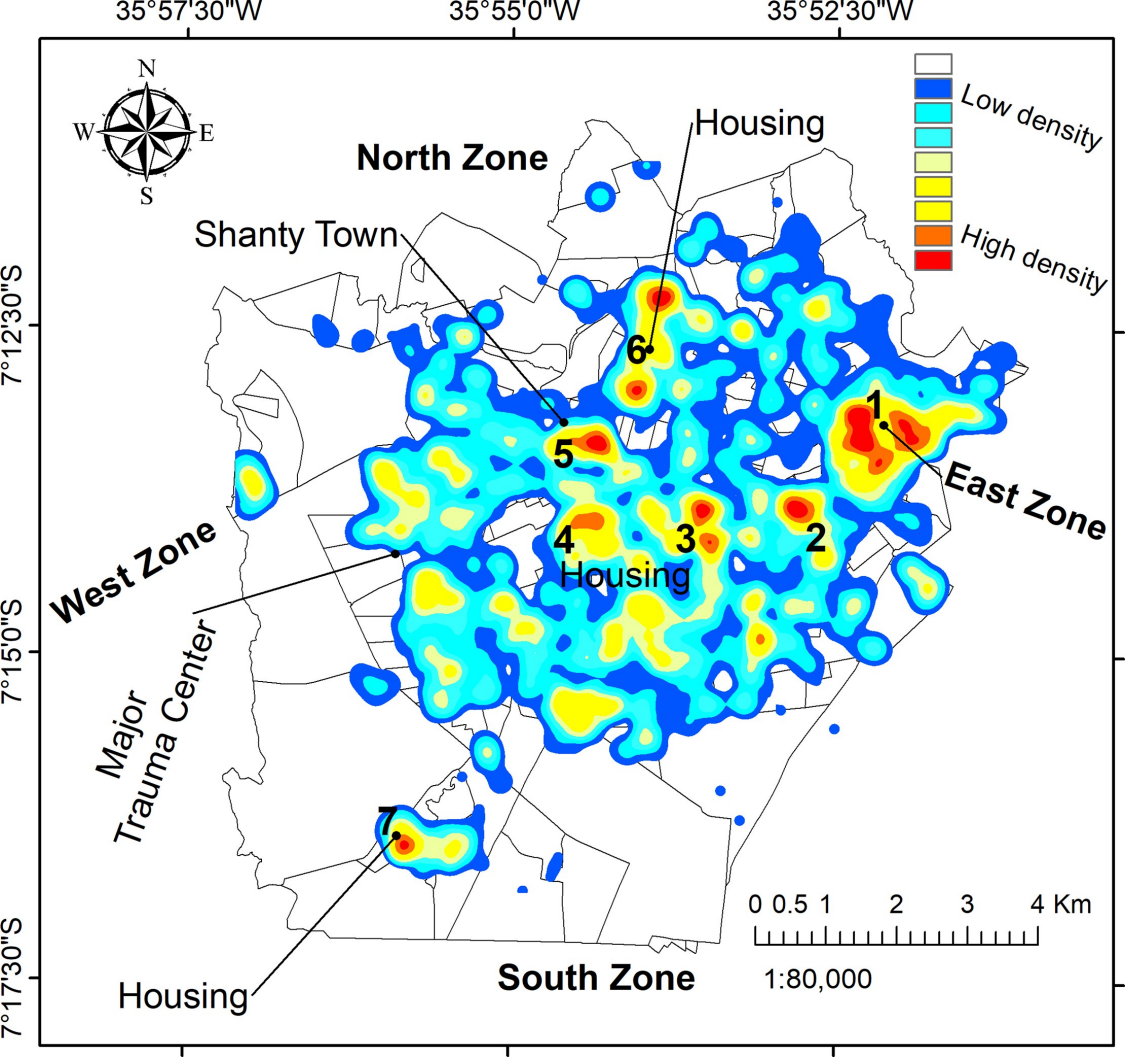
Density of all physical violence cases by victim’s household address. The seven most prominent clusters are numbered.

The use of GIS facilitated an exploration of violence and potential demographic covariates through a spatial lens. By mapping the place of residence for 2563 victims, we were able to delineate the areas of highest density, as shown in Fig 3. Seven prominent hotspots were identified: the East Zone, a large informal neighbourhood, and five distinct residential areas. The highest density of cases was found in the East Zone, while the West Zone and South Zone featured no prominent hotspots.

**Fig 4:**
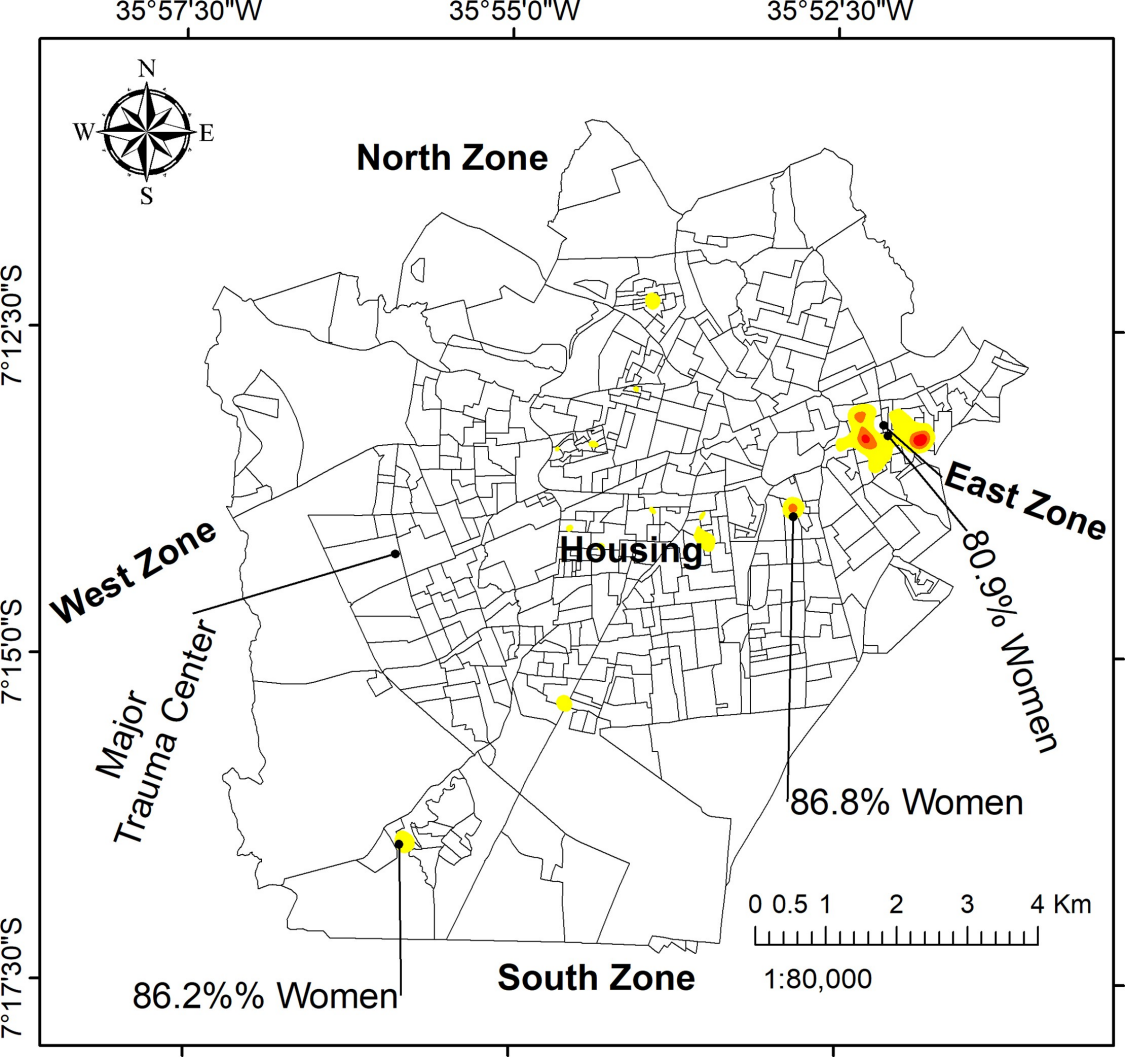
Density of domestic violence cases.

**Fig 5:**
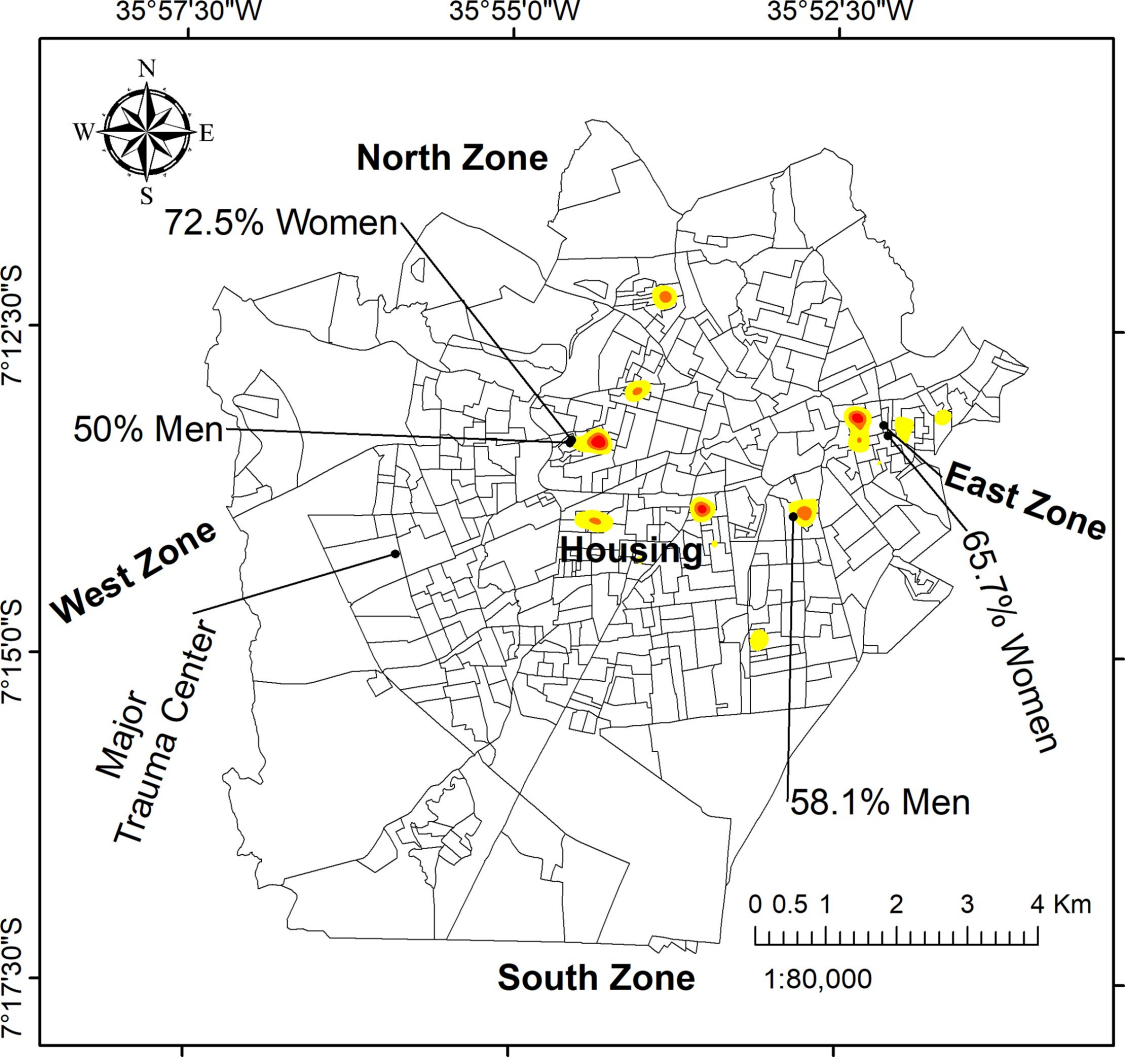
Density of community violence cases.

Domestic violence is tightly concentrated in the East Zone, while community violence tended to be more dispersed or scattered throughout the city, and in the informal settlements in the city centre (Figs 4 and 5).

The spatial distribution of male victim households were concentrated in hotspot 2 (near downtown), while female victim hotspots were more numerous, and particularly prominent in the East Zone. However, maps of the aggressor’s sex indicated no notable geographical pattern for neither male nor female, and there were no differences in the proportion of male and female aggressors throughout the study area.

**Fig 6:**
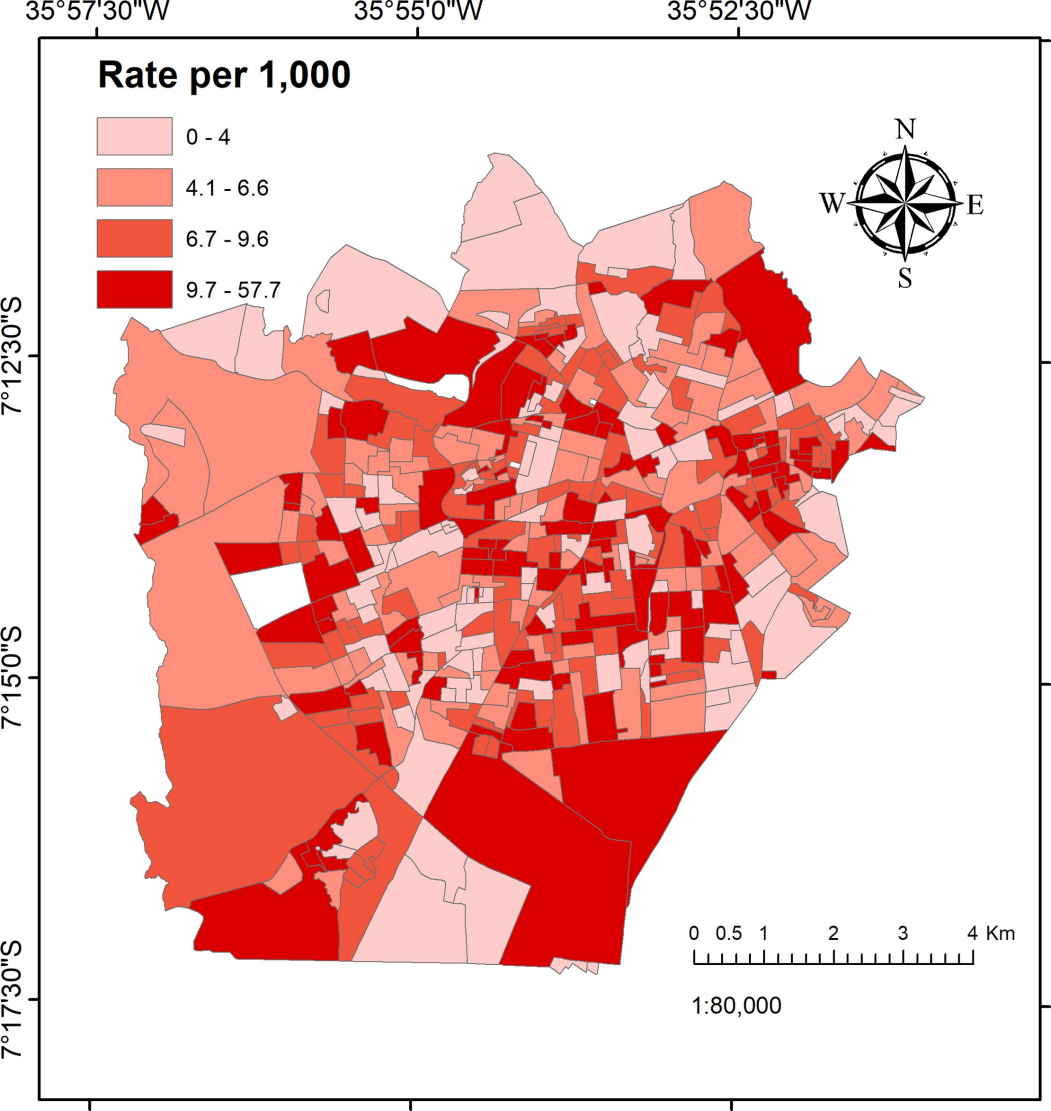
Rate of injured victims per 1,000 population, using a census tract map categorised by quartile.

**Fig 7:**
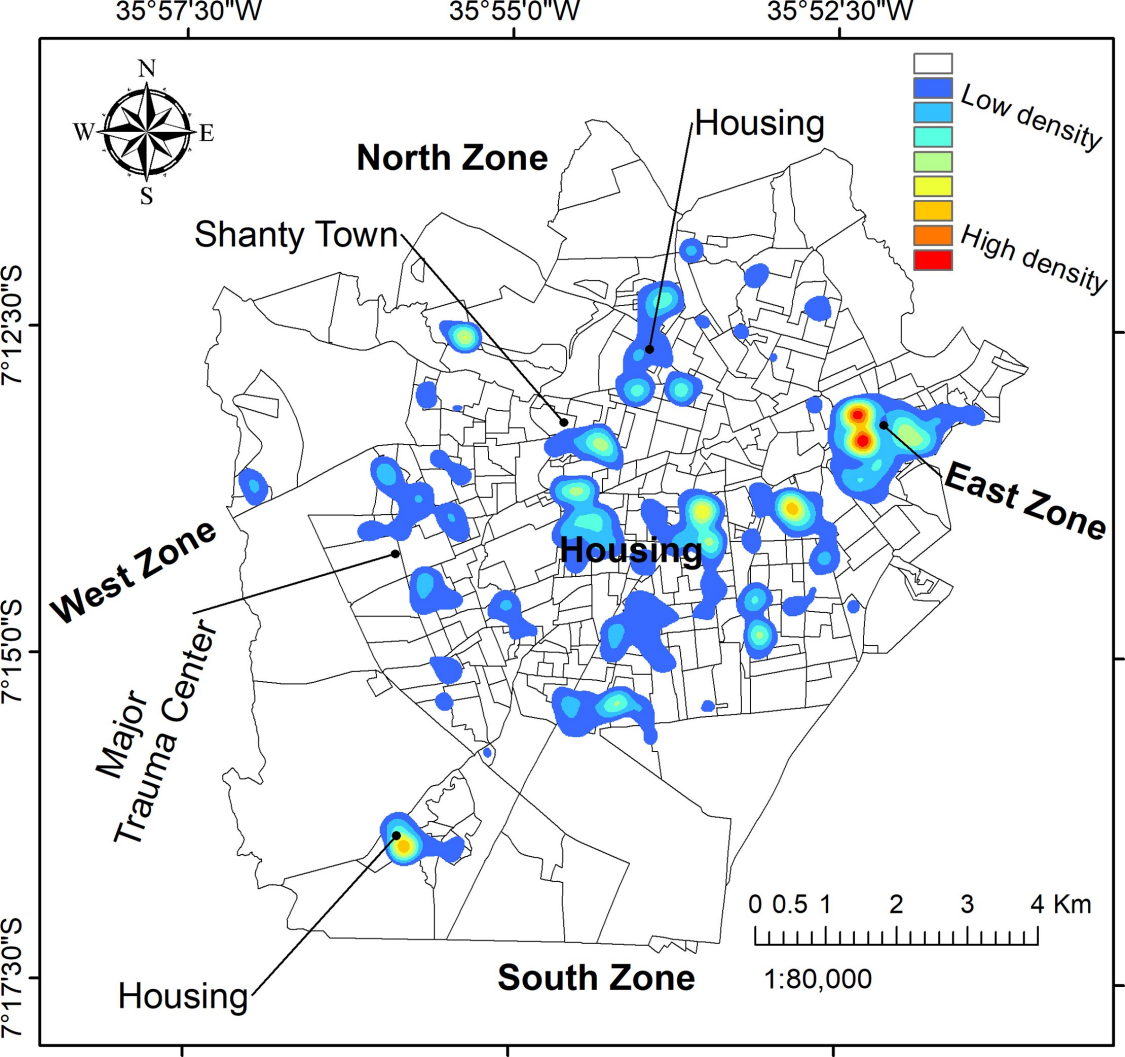
Local Moran’s I hotspots for all physical violence cases by victim’s household address.

The rate of injured victims per census tract is shown in Fig 6 and the Moran’s I clusters are shown in Fig 7. The Global Moran’s I score was I = 2.12 (*p*<0.001).

In Table 3, we highlight several variables of interest in the clusters and the non-hotspot area. The type of violence between clusters indicated a predominance of domestic violence, except for clusters 5 and 6. Clusters 1, 5, 6, and 7 featured the lowest family incomes and smaller interquartile ranges. Cluster 1 had numerous outlier points and cluster number 2 showed the greatest income variation.

**Table 3:** Distribution of selected variables for each cluster.

	Study Area							
	Not in hotspot	Cluster 1	Cluster 2	Cluster 3	Cluster 4	Cluster 5	Cluster 6	Cluster 7
**Age**								
mean	30.2	29.2	29.3	32.1	30.8	26.9	29.4	27.0
SE	0.3	0.7	1.5	1.4	1.5	1.4	1.0	1.6
Q1	21.0	19.0	21.0	20.0	20.0	19.0	21.0	19.5
median	28.0	27.0	27.0	30.0	28.0	24.0	28.0	25.0
Q3	37.0	35.0	38.0	41.5	43.0	34.0	36.0	32.0
**Victims’ Sex (%)**								
female	66.2	73.9	56.1	76.9	68.1	79.5	70.1	77.1
**Aggressor’s Sex (%)**								
male	70.1	68.0	68.8	69.3	67.1	60.8	61.7	69.8
**Type of Violence (%)**								
domestic	54.7	58.0	52.3	51.5	52.3	48.1	47.6	63.0
community	45.3	42.0	47.7	48.5	47.7	51.9	52.4	37.0
**Family Average-Income /census tracts**^**a**^ **(R$**^**b**^**)**								
mean	1,217.3	774.5	1,487.5	1,009.2	832.8	674.6	677.1	366.0
SE	23.6	31.6	107.6	30,8	20.3	90.6	36.8	10.6
Q1	623.9	554.9	660.6	829.8	719.3	409.3	515.2	331.1
median	903.0	650.0	958.0	894.1	710.1	457.4	549.1	331.1
Q3	1,459.4	793.0	2,562.0	1267.4	994.3	521.0	722.9	356.4

^a^Data from Brazilian 2010 census.

^b^Brazilian Reais (1$ ≈ 3R$ and 1€ ≈ 4R$).

## Discussion

This research focusses on both epidemiological and spatial characteristics of violence in an urban Brazilian population. This dual approach highlights socioeconomic drivers of injury and enabled us to differentiate between the spatial and aspatial characteristics of domestic and community violence.

Eight out of ten victims of domestic violence were women and the proportion of female victims was 3.2 times greater than man. This number is consistent with data from the Ministry of Health for the study area since 2009, when the country began to report an increase in violence against women [21]. Similar patterns was found in the USA, as the CDC reported significant higher lifetime prevalence for female (32.9%) than male (28.1%) [22]. Our results are consistent with the global trend of high lifetime prevalence of domestic violence against women victims [23–26].

The WHO study of domestic violence against women in ten countries, including Brazil, also revealed that the prevalence of physical violence in low- and middle-income countries was very high for both urban and rural areas [27]. Schraiber et al. reported the lifetime prevalence of violence to be 46.4% in São Paulo and 54.2% in Pernambuco (Northeastern), and that physical violence usually accompanied sexual and psychological abuse [28]. These two data far exceed the global estimate of 30%, and some factors are suggested to explain the reasons why female victims women are less likely to file criminal complaints and seek external help [6].

Sixty percent of the study population was between 19 and 39 years old, a cohort with 1.23 times greater odds of domestic violence than persons ages 0 to 18 (multiple regression). This may correspond with a previous National study identifying a spike in the rates of youth death by homicide [29]. Those victims ages 19–39 were more likely to be victims of domestic violence and young under 18 were slightly more victims of community, which may run contrary to the common belief that children and adolescents are more often injured in a familial context. Note, however, that 65% of the young population were child victims of domestic violence, compared to only 40% of adolescents. It is needed to consider that no statistical significance was found after multiple regression model for age-range, so this variable should be carefully interpreted at the present research.

A violent event during early childhood is known to affect emotional and social development and ability to succeed in school [30,31]. The most common familial risk factors are low socioeconomic status/poverty, antisocial parents, poor parent-child relation, broken home, separation from parents, abusive and neglected parents [32].

Regarding victims’ marital status, we observed very high odds of domestic violence for a person who lives with their partner but is not married, compared to single persons. This is concordant with a recent study in Portugal, which similarly found that non-married couples were more likely to report physical abuse, and on multiple occasions, compared to married couples [33].

The occupation status of victims showed that people who were not working had 1.35 times higher odds of domestic violence than wage earners, a significant result for the final model. The category *not working* included a large number of female homemakers, most of whom were victims of domestic violence, particularly by their partner. A recent Saudi study also explored impact of occupation on domestic violence risk, finding that unemployed women were more likely to be injured than students, teachers, and office staff [34].

In the present research, the main mechanism of injury was bodily force and included: punches; kicks; slaps; bite; pinches; hair pulling; attempt to strangle; scratches; elbow; headbutt; knee and push. Altogether, these mechanisms were responsible for the soft tissue injuries and did not cause life threatening injuries. However, it is expected that this type of physical violence can produce harmful effects for all ages, from the youth to elderly, with attendant loss of self-esteem, anxiety/depression, suicidal thoughts and post-traumatic disorders [35–37]

The vast majority of aggressors in this study were men, as holds true globally. This is particularly important because the direct and indirect effects of it violence can lead to disability and death on women [6]. In Brazil, sociocultural conventions may encourage or incentivise male aggression, resulting in sustained gender inequality and persistently high levels of violence against women [38]. There is some evidence that the incidence of female aggression is recently increasing, but may have different characteristics such as self- or family-defence, fear, and social control within a relationship [39].

The use of GIS enabled the identification of seven cluster regions across the study area. Indeed GIS enabled identification of spatial zones for prevention focus, an important policy intervention. The East Zone had the largest cluster and contained the majority density of victims (Fig 1). After controlling the hotspot analysis for population size (Fig 7), we can observe that the map is similar to the map of density in Fig 3. The similarity is especially high in the East Zone, clusters number 2, 3, and 7, which provide us that violence action prevention are urgent in these areas.

The East Zone region comprises seven neighborhoods, five of which have notable common characteristics according to the most recent Brazilian census in 2010 [18], such as a high population density (from 3530 to 13207 persons per km^2^) and high number of residents per household (3.4–3.8). Cubbin et al. (2000) found that a high proportion of crowded housing in the USA was associated with homicide [40]. Walker & Schuurman (2012) identified a correlation between clusters of violent injury and high-density residential areas and alcohol-serving establishments [10].

In the spatial analysis, we needed to consider that the population size could influence in the hotspot distribution. Indeed, we explored how the effect of population size can affect the results by calculating the number of injured victims per census tracts and estimating the rate per 1,000 population. After this analysis we could see that although the hotspots for violence were coincident with census tracts with high injury rate, and the hotspot map in Fig 7 proves that, as we can see a similar patterns comparing with the KDE analysis.

Conversely, this study found that areas with a high density of alcohol-serving establishments did not necessarily feature a high density of violent cases, for example, in the West Zone. Further analysis of the East Zone may explain why it features such a significant hotspot for violence. Through local knowledge of the study area, our team identified four key characteristics of this zone that may help to explain the observed concentration: 1) a lack of infrastructure and high levels of environmental disorder (e.g., pollution); 2) a high concentration of high-density housing; 3) many irregular and informal settlements and 4) among the lowest incomes in the city.

When categorized by type of violence, we identified some differences in the spatial pattern of incidence, for example, in cluster 2 most victims were male. This may be due to the fact that this region is well-known for its high rate of urban crime such as robberies and other potentially violent acts involving men. However, little differences in the spatial distribution of cases by victim sex were identified, perhaps obfuscated by the vast majority of cases involving female victims. A previous study in Vancouver did identify different patterns between male and female hotspots, though the underlying drivers of violence were varied and somewhat dissimilar to those identified in this study [11].

To describe differences between clusters we selected each hotspot and summarized variables of interest. The clusters generally exhibited similar distributions of age, victims and aggressor sex, and type of violence. However, we did observe that the average family income did vary between several clusters. The non-hotspot area had the second highest average income, compared to the hotspots. There is evidence that urban violence and homicide hotspots are associated with low income and high unemployment [19,20,40]. Although low-income coincided with hotspot areas, this alone does not explain all cases of violence; some violent events occur among middle- and high-income families, which may have been obscured in our data, due to the excessively high clustering of incidence in low-income areas.

According to the Global Status Report on Violence, the communities with high concentration of poor people tend to have residential instability, difficulties to stablish common values and norms, and these social disorganization combined with economic disadvantage create conditions for high violence rate, social marginalization and also poor physical and mental health [11]. The relationship between violence and poverty is more visible for community violence related to crimes since the 70’s we have evidence that poverty is associated with income inequality in violent crimes, including homicides and assaults [41]. Some authors have already provided a conceptual framework using an ecological model that confirm the violent crimes is well associated with social deprivation, including income inequality and low social capital [42].

The income data used in this study were aggregated census data from 2010; as such, the income values reflect neighborhood averages, and do not necessarily indicate a victim’s family income. The victim location was recorded only as their residential address, and not necessarily the location at which the incident took place. These are common limitations for geographical studies. Crucially, the underreporting of violence, particularly for female victims, is well documented and suggests that the figures released in this study are significantly lower than the true numbers. We were unable to map some victims due to missing or erroneous address data. According to the City Hall, there are more than 400 unnamed streets in the study area, posing a significant challenge for geospatial analysis. Despite the limitations aforementioned, we suggest that the missing address seems to not have affect the quality data, once that we successfully geocoded 72.0% of the total injuries victims. It is possible that including the 996 missing address, the hotspots could slightly change position, but we suppose that this would not affect global distribution of violence.

## Conclusion

In conclusion, we described epidemiological and spatial patterns for interpersonal physical violence in a Brazilian medium-sized city. Women, people living together, bodily force injuries, and male aggressors were the most prominent significant factors associated with violence in a domestic context. When we look at the epidemiological findings spatially, it becomes evident that the hotspots areas for violence are coincident with the poorest city regions. This spatial identification of the overlap between hotspots and family income are the basis for future injury prevention strategies.

## Supporting information

S1 File Data. Data Violence table in excel showing regional locations and frequency of violent events.
(XLS)
